# The immunological interface: dendritic cells as key regulators in metabolic dysfunction‐associated steatotic liver disease

**DOI:** 10.1002/1873-3468.15072

**Published:** 2024-12-12

**Authors:** Camilla Klaimi, WanTing Kong, Camille Blériot, Joel T. Haas

**Affiliations:** ^1^ Univ. Lille, Inserm, CHU Lille, Institut Pasteur de Lille, U1011‐EGID Lille France; ^2^ Gustave Roussy, INSERM U1015 Villejuif France; ^3^ Gustave Roussy, CNRS UMR9018, Metabolic and Systemic Aspects of Oncogenesis for New Therapeutic Approaches, Université Paris‐Saclay Villejuif France; ^4^ Institut Necker Enfants Malades, CNRS, INSERM, Université Paris Cité France

**Keywords:** dendritic cells, immunometabolism, liver, MASLD, regulation

## Abstract

Metabolic dysfunction‐associated steatotic liver disease (MASLD) refers to a broad spectrum of conditions associating fat accumulation in the liver (steatosis) with varying degrees of inflammation (hepatitis) and fibrosis, which can progress to cirrhosis and potentially cancer (hepatocellular carcinoma). The first stages of these diseases are reversible and the immune system, together with metabolic factors (obesity, insulin resistance, Western diet, etc.), can influence the disease trajectory leading to progression or regression. Dendritic cells are professional antigen‐presenting cells that constantly sense environmental stimuli and orchestrate immune responses. Herein, we discuss the existing literature on the heterogeneity of dendritic cell lineages, states, and functions, to provide a comprehensive overview of how liver dendritic cells influence the onset and evolution of MASLD.

## Graphical abstract

Metabolic dysfunction‐associated steatotic liver disease (MASLD) affects nearly one‐third of the global population and poses a significant risk of progression to cirrhosis or liver cancer. Here, we discuss the roles of hepatic dendritic cell subtypes in MASLD, highlighting their distinct contributions to disease initiation and progression, and their potential as therapeutic targets for preventing advanced liver complications.

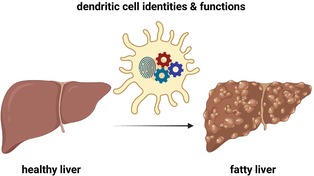

## Abbreviations

**CD**, cluster of differentiation

**CDAA**, choline‐deficient‐amino acid diet

**cDCs**, conventional DCs

**DC**, dendritic cells

**DTA**, diphtheria toxin subunit A

**DTR**, diphtheria toxin receptor

**HFD**, high‐fat diet

**IFN‐I**, type‐I interferon

**IL**, interleukin

**KC**, Kupffer cell

**MASH**, metabolic dysfunction‐associated steatohepatitis

**MASLD**, metabolic dysfunction‐associated steatotic liver disease

**MCD**, methionine‐ and choline‐deficient diet

**MHC**, major histocompatibility complex

**NAFLD**, nonalcoholic fatty liver disease

**NASH**, nonalcoholic steatohepatitis

**OXPHOS**, oxidative phosphorylation

**PAMP**, pattern‐associated molecular pattern

**PBMC**, peripheral blood mononuclear cell

**PC**, phosphatidylcholine

**pDCs**, plasmacytoid DCs

**PD‐L1**, programmed cell death ligand 1

**PGE2**, prostaglandin E2

**RA**, retinoic acid

**scRNAseq**, single cell RNA sequencing

**TCA**, citric acid cycle

**TGFβ**, transforming growth factor

**T**_**H**_, T helper cell

**TLR**, Toll‐like receptor

**Treg**, regulatory T cell

**T**_**RM**_, memory T cell

**VLDL**, very‐low‐density lipoprotein

**WD**, western diet

**ZBTB46** Zinc Finger and BTB Domain‐Containing Protein 46

Since their identification almost 50 years ago awarded by the Nobel Prize in Physiology‐Medicine 2011, dendritic cells (DCs) are now recognized to play important and diverse roles in many different diseases. They are at the interface between innate and adaptive immunity, showing expression of a broad panel of pattern recognition receptors and very potent activity for antigen processing and presentation. Recent studies have demonstrated the role of both intra‐ and extracellular metabolic factors in tuning immune cell—including DC—functions. This has opened the new field of research of *immunometabolism*, which focuses broadly on the interactions between metabolism and immunity and how both systems are impacted in the context of diseases.

In the present Review, we will focus on the role of hepatic DCs in normal physiology and current knowledge about their role in the pathophysiology of metabolic diseases, especially metabolic dysfunction‐associated steatotic liver disease (MASLD). Much of what is known is derived from animal studies, but we will indicate parallels in human pathology as much as possible based on available data. We aim to highlight innovative lines of research rendered possible by recent and emerging technological developments.

## Half a century of dendritic cell history

**Fig. 1:**
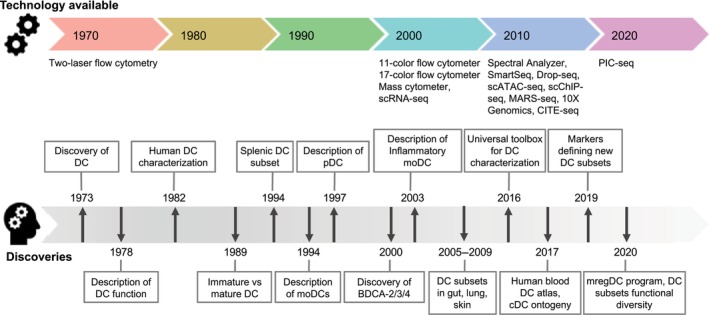
Evolution in DC characterization alongside technological advances over the years.

As their name indicates, DCs were first introduced based on their morphology only, harboring typical “tree‐like” structures (=dendrites) when microscopically observed in tissues, in addition to other features [1]. Their functional description only came later, with the reported lower endocytic capability as compared to macrophages and the absence of antigen binding, differentiating them from lymphocytes [2, 3]. It is useful to remember that, at this time, the concept of antigen‐presenting cells was not established yet [4, 5, 6] and several years passed before their now well‐established role in stimulating lymphocyte proliferation was documented (Fig. 1) [7]. Indeed, DCs are often considered as the bridge between innate and adaptive immune responses, because of their ability to capture and process antigens in peripheral tissues, before migrating to lymph nodes and presenting the antigens on major histocompatibility complex (MHC) molecules to prime T cells. Briefly, DC‐mediated activation of T cells is a multistep process requiring complementary signals [8]: peptide presentation on MHC molecules of DCs, co‐stimulation of T cells through interaction of molecules such as T cell CD28 with DC CD80/86 and cytokine production. DCs can also provide post‐priming signals to T cells for sustenance of activation and formation of tissue‐resident memory T cells (T_RM_) [9].

The DC family slowly expanded over the years and they are now mainly categorized as plasmacytoid DCs (pDCs) and different subsets of conventional DCs (cDCs) [10, 11].

While pDCs are commonly identified as an entirety with an exceptional type‐I interferon (IFN‐I) and pro‐inflammatory cytokine production ability upon activation [12, 13], cDCs are reported to be proficient in antigen presentation and priming of naive T cells.

Phenotypically, these different populations can be easily identified. First, the most common surface markers used for DC identification remain CD11c and MHCII in mice or HLA‐DR in humans, pDC expressing them at a lower level as compared to cDCs. In addition, the transcription factor Zinc Finger and BTB Domain‐Containing Protein 46 (ZBTB46) has been reported to be highly expressed by cDCs and their progenitors, but not by other immune populations including pDCs and monocytes [14]. Conversely, pDC‐specific markers such as SiglecH or CD317 can be used [15, 16]. cDCs can be then split into two main populations, cDC1 and cDC2, with many markers available to discriminate them, cDC1 expressing for example genes such as *CADM1* and *XCR1*, while cDC2s are commonly identified thanks to their expression of CD1c, CD11b, or more recently, CD172a [17, 18]. Of note, we can often read that DC1 help in differentiating T helper 1 (T_H_1) cells while DC2 are more directed toward T_H_2 differentiation, but it has to be stated that these DC1/DC2 refer to outdated nomenclature and should not be misinterpreted as the current cDC1/cDC2 one, for which such functional dichotomy has not been observed [19]. Finally, within the cDC2s, another level of heterogeneity has been reported with the coexistence of several subpopulations based notably on the expression of CD5, CD14 and CD163 [20, 21, 22]. Of note, all cDCs are able to migrate to lymph nodes to present antigens to naive T cells and their phenotype can in this case be slightly altered, for instance with the notable acquisition of CCR7 [23, 24]. Migrating CCR7^+^ cDCs correspond mostly to a reversible state which can arise from both cDC1 or cDC2, and should therefore not be confused with the different lineages pDCs, cDC1s and cDC2s described above. To complete the description of the DC family, one should also mention skin Langerhans cells described more than 150 years ago [25] which exhibit features of both macrophages and DCs. Finally, monocyte‐derived DCs, referring to cells originating from circulating monocytes and not dendritic cell precursors, can adopt a DC‐like phenotype in some inflammatory settings, including the capacity to migrate to lymph nodes through the CCR7 pathway [26, 27] as well as the expression of the canonical cDC marker ZBTB46 [28]. This last observation illustrates that distinct immune populations such as monocytes and dendritic cells can converge in terms of phenotype and functions, rendering them indistinguishable with conventional tools such as flow cytometry. In this context, fate‐mapping tools combined with high‐dimensional analysis such as single cell RNAseq are necessary.

DC ontogeny has considerably evolved in last years and still constitutes an active field of research [29]. The identification of a common FLT3^+^ precursor for pDCs and cDCs in the bone marrow more than 15 years ago led to the DC recognition as a unique lineage, distinct from other immune cells [30, 31]. Since then, pDC‐specific progenitors have been identified and their inclusion in the DC family is currently discussed [32, 33, 34, 35]. Additionally, from bone marrow progenitors, cDCs egress under the form of pre‐cDCs, with already a pre‐imprinted program of pre‐cDC1 or pre‐cDC2, and reach the peripheral tissues from circulation to achieve their maturation [36]. However, pre‐DC homing into tissues and notably how a given cell will “decide” to migrate to the liver or any other organ remains an open question. Another related question is how a given tissue will imprint DC biology with tissue‐specific identities and functions, similar to what is known for macrophages for example [37, 38]. An illustrative example is the capacity for small intestinal cDC to recruit gut‐tropic T cells thanks to their imprinting by retinoids, a function not shared by cDCs from other tissues [39].

Finally, the cDC subtypes have closely related functions, but differ in their repertoire of pattern recognition receptors (PRR) and their propensity to activate specific T cell responses [40]. Besides TLR2 and TLR4 highly expressed by all subpopulations of cDCs, cDC1 in both mice and humans express high levels of TLR3, which is an endosomal sensor for double‐stranded RNA and favors cross presentation to CD8^+^ T cells. The receptor DNGR‐1 (Clec9A) is also cDC1‐specific and recognizes polymerized F‐actin from dead cells. Conversely, cDC2s express high levels of TLR5, which recognizes bacterial flagella, and various viral receptors such as TLR7 (single‐stranded RNA), RIG‐I and MDA‐5. This differential expression of PRR shows that even if all subpopulations of cDCs are well equipped to sense and mount a response to various intruders, small differences exist between them, allowing to refine such responses.

## The spectrum of metabolic dysfunction‐associated fatty liver diseases

Metabolic dysfunction‐associated steatotic liver disease (MASLD, previously referred as nonalcoholic fatty liver disease or NAFLD), is a chronic, progressive liver disease, which now affects nearly 30% of the global adult population, with discrepancy around the world from 25% in Western Europe to 44% in Latin America [41]. In early stages, MASLD is characterized by accumulation of fat, mainly in the form of triglycerides, in hepatocytes without other signs of hepatic damage. Over time, steatosis leads to impaired hepatocyte function which is associated with both recruitment of systemic immune cells to the liver compartment and activation of liver‐resident immune cells. When histological evaluation indicates the combined presence of steatosis, lobular inflammatory foci and hepatocyte ballooning, an individual is diagnosed with metabolic dysfunction‐associated steatohepatitis (MASH, previously called NASH). MASH increases the risk of fibrosis, hepatocellular carcinoma, development of type‐2 diabetes, cardiovascular diseases, and cirrhosis and is now the leading cause of liver transplantation [42, 43]. During the progression of MASLD, the liver undergoes profound structural changes which impact normal tissue homeostasis. The first appearance of steatosis occurs in hepatocytes in the pericentral region and progresses outward toward the periportal regions as the steatosis severity increases [44]. In parallel, transient episodes of hepatocyte necroinflammation occur, leading to cycles of recruitment of immune cells from the periportal zone into the lobular regions [45]. In later stages, activation of stellate cells in collagen‐producing myofibroblasts will drive marked collagen deposition, which physically deforms the lobular structure [46].

Much of what we understand about MASLD pathophysiology comes from animal models, which can differ widely in their mechanisms of disease induction and the associated metabolic phenotype. High‐fat diet (HFD), for which usually 60% of energy comes from fat, induces obesity and insulin resistance and often only mild steatosis up to 16 weeks of diet. When combined with high sucrose (high fat high sucrose) and added cholesterol (Western diet), this can drive more marked steatosis and stronger activation of hepatic inflammation as a result of enhanced lipogenesis and excess cholesterol. Another widely used model of MASLD in mice is the methionine‐ and choline‐deficient (MCD) diet or choline‐deficient‐amino acid defined (CDAA) model. This works by strongly reducing the availability of hepatic phosphatidylcholine (PC), thereby inhibiting hepatic very‐low‐density lipoprotein (VLDL) output and rapidly inducing steatosis. Both MCD and CDAA diets also induce oxidative stress by affecting normal amino acid levels for glutathione synthesis and mitochondrial function. Especially in MCD diet, there is marked loss of body mass, with a strong reduction of adipose tissue. Considering the close association between obesity and MASLD, these types of models have been particularly criticized for their physiological relevance, though many of the pathophysiological features are faithfully recapitulated. Finally, to favor fibrosis development particularly in C57BL6/J mice, a common method is chemical induction by carbon tetrachloride (CCl_4_) injections in combination with the Western‐type diet models discussed [47].

Nevertheless, it is essential to remember that human MASH refers to a spectrum of conditions and none of the aforementioned models should be used as a universal one [48]. To gain fruitful insights and generate robust data, an efficient strategy is often to combine few of them and ideally cross‐validate with observations on patient samples.

## Hepatic DCs are tolerogenic in healthy conditions

Despite its perception as primarily a metabolic organ, the liver contains a full complement of resident and infiltrating immune cells and can also be viewed as an immunological organ [49]. For example, Kupffer cells (KC), which are yolk‐sac‐derived resident hepatic macrophages constitute the major hepatic immune cells and the largest resident tissue macrophage population in the body. Conversely, dendritic cells are by far less frequent, representing a very minor fraction of hepatic immune cells at steady state (~ 1–5% of all immune cells), but with key functions when considering MASLD pathophysiology.

Due to its constant exposure to food toxins, by‐products of the microbiota and other potentially inflammatory stimuli, a healthy liver must preserve its homeostatic activity through immune tolerance [50]. This is primarily achieved by the secretion of immune mediators such as interleukin‐10 (IL10) [51], transforming growth factor (TGFβ), retinoic acid (RA) [52], and hepatocyte growth factor [53]. In the liver, the antigen‐presenting cells mediate immune homeostasis, for instance, KCs induce immune tolerance via their capacity to express programmed cell death ligand 1 (PD‐L1), implicating IL10 and prostaglandin E2 (PGE2) secretion and mediating regulatory T cell (Treg) activation [54]. Hepatic DCs typically display a tolerogenic immature phenotype [55], characterized by PGE2 and IL10 secretion to inhibit T cell proliferation, promote apoptosis of activated T cells, and generate Treg and Th2 cells [56]. In addition, tolerogenic hepatic DCs express low levels of major histocompatibility complex I and II (MHCI and MHCII, respectively) and therefore provoke impaired CD4^+^ T cell and CD8^+^ T cells proliferation and responsiveness [57, 58].

**Fig. 2:**
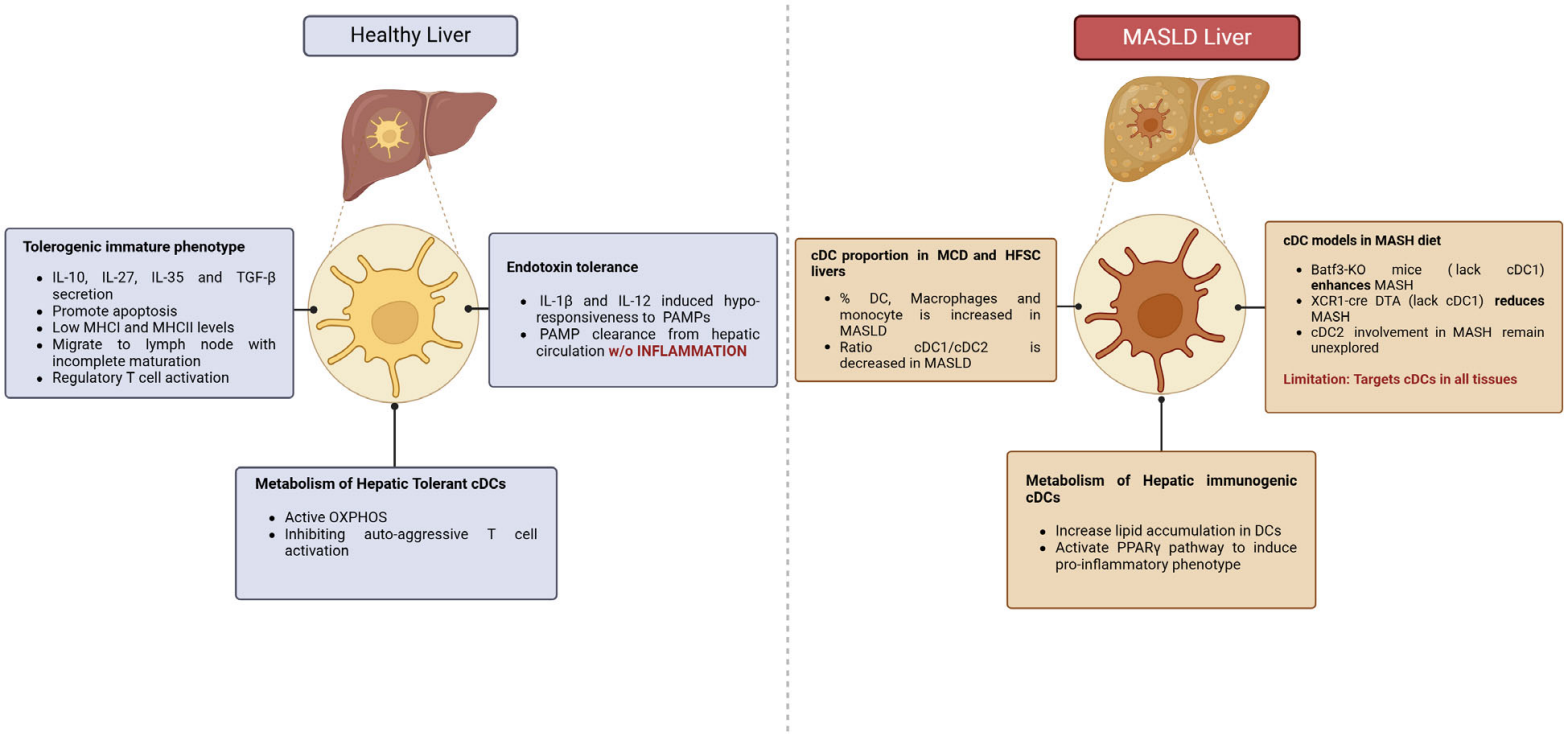
Comparison between cDC identities and functions at homeostasis and during MASLD. Main features of cDCs are indicated in homeostasis or in diseased state.

Interestingly, hepatic DCs are not as phenotypically and metabolically mature as their splenic counterparts. They exhibit a persistent behavior known as ‘endotoxin tolerance’ [56], which is characterized by an hypo‐responsiveness to pattern‐associated molecular patterns (PAMPs). This behavior is mediated by suppressors of TLR activity such as IL1β and NF‐kB, despite displaying intact scavenger function. Moreover, these cells increase the expression of IL1β, IL12B, and IL6, which favor PAMP clearance from hepatic circulation without inducing inflammation [59]. Together, these pieces of evidence indicate that hepatic DC have specific intracellular mechanisms to maintain a tolerogenic phenotype and a high concentration of anti‐inflammatory enhancers in the liver. Of note, we want to emphasize that such tolerogenic properties of hepatic DCs have to be considered in healthy conditions. Indeed, and as the other DCs in the organism, they are able to be activated in various conditions of challenge, from sterile inflammation to viral infections [60] and of course in the context of metabolic challenges (Fig. 2).

## Metabolic reprogramming of hepatic dendritic cells during MASH

It is becoming increasingly evident that intra‐ and extracellular metabolic signals play an important role in regulating immune cell functions [61]. The best described immunometabolic response in myeloid cells is the activation of glycolysis in response to TLR activation [62, 63, 64]. This response was actually first described one century ago by Otto Warburg by measuring lactate production in activated peripheral blood mononuclear cells (PBMCs), and is now widely referred as the “Warburg effect” [65]. With the advent of scRNAseq and metabolic tracer studies, it is now clear that this “glycolytic shift” is only a small fraction of the diversity of the metabolic changes that occur in immune cells in general and cDCs in particular [66]. Quiescent DCs metabolize glucose to pyruvate which mainly enters in the citric acid cycle (TCA) through the form of acetyl‐CoA. An active TCA cycle in quiescent DCs produces energy and activate oxidative phosphorylation (OXPHOS), which is a key pathway for maintaining DCs inactivated and sustaining their survival [67]. Thus, DCs sustain immune tolerance by inhibiting auto‐aggressive effector T cells activity and inducing T cell regulatory program [68]. Tolerogenic program is maintained in DCs by IL10, IL27, IL35, IL37 or TGFβ signaling, but also by circulating molecules such as vitamins A or D3 [69, 70, 71].

As mentioned earlier, healthy hepatic DCs exhibit a more tolerogenic phenotype than the ones in other tissue. In the context of MASLD, the increase of their lipid content activates cDCs notably through the *Pparg* pathway, resulting in a more pro‐inflammatory phenotype [72]. Reversely, inhibition of lipogenesis with C75 reduced the lipid content of cDCs and reduced their immunogenicity in a cross‐presentation assay. Therefore, with this study, authors suggest that the lipid content directly shapes cDC functions, lipid‐laden cDCs being much more immunogenic, while lipid‐low cDCs remained mostly tolerogenic [72]. Very interestingly, this observation could be MASLD‐specific, as an opposite observation has been made in cancer‐associated cDCs, where cDCs with increased amounts of triglycerides displayed lower immunogenic capabilities [73].

Of note, an unbiased analysis of the reference *Tabula Muris* dataset revealed that the different cDC subsets displayed distinct metabolic profiles, suggesting a differential metabolic reprogramming [74]. In this analysis, migratory CCR7^+^ cDC displayed high expression of genes involved in the cholesterol biosynthetic pathway, and treating mice with statins, which block cholesterol biosynthesis, was associated with reduced cDC migration to lymph nodes. In the context of cancer, acidic extracellular pH activates SREBP2, a master regulator of cholesterol biosynthesis, and targeting SREBP2 activity in cDCs enhanced anti‐tumor immune response [75]. Considering the status of statins as a first‐line treatment for hypercholesterolemia and the close association between MASLD and dyslipidemia, it remains to be deciphered how statin‐based therapies may affect cDC functions in MASLD.

## cDC subtypes and their functions in MASLD

Early studies of DC function in liver diseases demonstrated distinct roles in the development of MASLD. First, a 2–3 fold expansion of hepatic immune cells per gram of tissue was documented in the different models of MASLD, highlighting inflammation and the response of the immune system to liver challenge. The proportion of DCs, macrophages and inflammatory monocytes increased, while B cell proportion decreased [76].

More recently, this observation has been further dissected with scRNAseq, revealing a transient increase in both cDC1 and cDC2 populations [77] in the MCD model, though we have previously reported a relative decrease in cDC1 (as a proportion of CD45%) in a Western diet model [78]. Still, in several reports, including our own, the ratio of hepatic cDC1 to cDC2 is decreased and the proportion of circulating blood cDC1 is also decreased in MASH patients [77, 78, 79].

The specific changes in these populations suggested a key role in the development of MASLD, which has also been partially addressed. For example, clodronate‐mediated monocyte and macrophage depletion prior to dietary challenge revealed protection from liver damage in MCD [80] and Western diet [81] models. Conversely, DC depletion in CD11c‐Diphteria Toxin Receptor (DTR) transgenic mice enhanced both hepatic inflammation and fibrogenesis in the MCD model [76]. Though these studies used relatively blunt tools to investigate immune cell functions in MASLD pathogenesis, they clearly highlight opposite functions of hepatic DC and macrophages. Interestingly, Western diet‐induced metabolic dysfunction can also impact bone marrow myeloid progenitors [82], DC‐poesis being increased in MCD‐fed mice [77]. In a pure fibrosis model of CCl_4_ injections, Sutti *et al*. [83] have shown that hepatic DCs increased inflammatory cell recruitment through the fractalkine (CX_3_CL1) pathway, and that fractalkine itself contributed to the maturation and expansion of a CX_3_CR1^+^ cDC2 population. Together, these effects could lead to the replenishment of resident cells with those from circulation displaying a more pro‐inflammatory phenotype, thereby amplifying hepatic damage and MASLD development.

New tools and models have allowed us to more carefully assess different cDC population functions in MASLD. The Batf3‐KO mice, which lack cDC1 at steady state, show enhanced steatosis and inflammation on a high sucrose diet but not the MCD model [84]. Authors showed this phenotype could be attenuated with adoptive transfer of bone marrow‐derived Clec9A^+^CD103^+^ cDC1, with reduced hepatic *Ccl2* expression and reduced monocyte recruitment. Surprisingly, a similar study using Xcr1‐Cre targeted DTA to deplete cDC1 revealed protection of MASLD in MCD‐fed mice, indicating a rather pro‐inflammatory role for cDC1. Indeed, Xcr1‐DTA mice displayed decreased markers of hepatic damage on MCD diet and decreased presence of effector memory CD8^+^ T cells [77]. The discrepancies in these results could arise from several reported differences in Batf3‐KO and Xcr1‐DTA models. Increased intestinal permeability has been reported in the Xcr1‐DTA model [85], but not the Batf3‐KO mice [86]. Additionally, cDC1 can arise in Batf3‐KO mice in certain inflammatory contexts [87], and *Batf3* also affects CD8^+^ memory T cell [88] and Treg functions [89], thus complicating the interpretation of the global Batf3‐KO studies. Conversely, Xcr1‐Cre is reported to have high germline recombination rates [90]. It is unclear what effect that may have in the DTA model and potentially complicates the interpretation of studies using Xcr1‐targeted gene disruption.

So far, the role of cDC2 in MASLD has not been precisely investigated, notably due to the lack of specific genetic tools to target this population. This may reflect the observed functional heterogeneity of cDC2 as well as the similarities in marker gene expression between cDC2 and monocytes. Another limitation for genetic models is that they impact cDCs in every tissue, and there is currently no way to specifically target only hepatic DCs.

## Interactions with other immune cells

As highlighted above, cDCs coordinate hepatic immune responses in MASLD directly and through recruitment and activation/polarization of other cell types. A key parameter to account for when considering their functions is the liver zonation. Indeed, despite of its apparent homogeneity, the liver exhibits a spatial division of labor, with hepatocytes performing amino acid catabolism and β‐oxidation mostly located in the portal zone, while the ones dedicated to glycolysis and lipogenesis are mostly found in the central zone [91]. Similarly, endothelial cells [92] and immune cells [93, 94] have distinct functions according to their location in the liver. Indeed, environmental signals including chemokine concentration, oxygen and nutrient availability and cellular partners vary based on this parameter, and will potentially imprint cDCs with distinct identities and functions. The current rise in spatial biology including transcriptomics, proteomics, lipidomics and interactomics should clarify soon the differences between the distinct subpopulations of hepatic cDCs and how these evolve during metabolic and inflammatory challenges such as MASLD.

For now, we and others have reported expansion of CD8^+^ T cells, especially a specific population of so‐called “auto‐aggressive” CXCR6^+^ CD8^+^ T cells thought to be responsible for liver injuries in MASH [95]. Whether these cells are antigen restricted, and if cDCs are responsible for the presentation of this antigen, is not yet fully established. Dudek *et al*. proposed a cDC‐independent mechanism whereby increased IL15 would lead to the expansion of these cells in an environment rich in acetate from apoptotic cells and induce the auto‐aggressive phenotype [95]. Moreover, Henning *et al*. reported that liver cDCs from MCD‐fed mice had a higher capacity to induce antigen‐restricted CD4^+^ T cell proliferation and production of Th1, Th2, and Th17 cytokines, while CD8^+^ T‐induction by control and MCD‐derived cDCs was not modified. Also, Van der Zande *et al*. [96] demonstrated that LKB1‐AMPK/SIK signaling pathway in CD11c^+^ DCs limits Th17 polarization in HFD‐fed mice, playing a protective role in MASLD. Furthermore, DCs interact with NK cells in order to enhance their activity; one example mentioned by Thomas *et al*. showed that IL15 trans‐presentation on DC‐activated NK cells primed by poly (I:C) [97].

## Concluding remarks

DCs have joined the family of immune cells 50 years ago and have since emerged as major regulators of many diseases. Their establishment as a distinct lineage took time and is still currently an active field of research, but it is now clear that DCs have their own progenitors, identities, phenotypes, and functions rendering them unique within the immune system. Nevertheless, they come in different flavors: pDCs, cDC1s, subpopulations of cDC2s, pre‐cDCs, and migratory DCs.

Here we have focused on the relationship between cDCs and MASLD, voluntarily putting aside pDCs, which are a distinct lineage, and should therefore be considered independently of cDCs. Due to their antigen presentation and migratory capacities, cDCs orchestrate adaptive immune responses to liver damage observed in the context of MASLD. For this, they undergo transcriptomic and metabolic rewiring in the stressed liver and report the danger to the central immune system. But it is also becoming evident that cDCs have a local effect within the liver, independent of adaptive immune cells, even if we are still lacking convincing proof to validate this hypothesis.

Nevertheless, the burst in single cell transcriptomics over the last decade offers now tools to precisely assess cDC identity and functions in the different models of MASLD available to the community. With the current emergence of spatial transcriptomics and lipidomics, we will for sure refine our view of cDC biology, notably by deciphering local interactions of the different subpopulations of cDCs with other hepatic cells and clarifying lipid exchanges between them.

While rodent models are obviously valuable to dissect basic mechanisms of the pathophysiology of MASLD, these models are by definition limited, and do not fully recapitulate the spectrum of human MASLD. Consequently, these studies should be coupled with clinical observations and analysis of patient samples. With obesity emerging as the major epidemic worldwide for this century, and considering that MASLD impacts 1 out of 3 adults, the range of possibilities is more open than ever, and it is up to us now to pursue our efforts to better understand cDC functions in MASLD.

## Conflict of Interest

The authors have no conflicts of interest to declare.

## Author contributions

CK and WK wrote the manuscript, CB and JTH conceptualized the review.
